# Bringing Together Robotics, Neuroscience, and Psychology: Lessons Learned From an Interdisciplinary Project

**DOI:** 10.3389/fnhum.2021.630789

**Published:** 2021-03-29

**Authors:** Olga A. Wudarczyk, Murat Kirtay, Anna K. Kuhlen, Rasha Abdel Rahman, John-Dylan Haynes, Verena V. Hafner, Doris Pischedda

**Affiliations:** ^1^Department of Psychology, Humboldt-Universität zu Berlin, Berlin, Germany; ^2^Adaptive Systems Group, Department of Computer Science, Humboldt-Universität zu Berlin, Berlin, Germany; ^3^Faculty of Philosophy, Berlin School of Mind and Brain, Humboldt-Universität zu Berlin, Berlin, Germany; ^4^Bernstein Center for Computational Neuroscience, Charité – Universitätsmedizin Berlin, Corporate Member of Freie Universität Berlin and Humboldt-Universität zu Berlin, Berlin, Germany; ^5^Milan Center for Neuroscience, Milan, Italy

**Keywords:** interdisciplinarity, human-robot interaction, social robotics, collaboration, robotics, social intelligence, cognitive neurorobotics, diversity

## Abstract

The diversified methodology and expertise of interdisciplinary research teams provide the opportunity to overcome the limited perspectives of individual disciplines. This is particularly true at the interface of Robotics, Neuroscience, and Psychology as the three fields have quite different perspectives and approaches to offer. Nonetheless, aligning backgrounds and interdisciplinary expectations can present challenges due to varied research cultures and practices. Overcoming these challenges stands at the beginning of each productive collaboration and thus is a mandatory step in cognitive neurorobotics. In this article, we share eight lessons that we learned from our ongoing interdisciplinary project on human-robot and robot-robot interaction in social settings. These lessons provide practical advice for scientists initiating interdisciplinary research endeavors. Our advice can help to avoid early problems and deal with differences between research fields, prepare for and anticipate challenges, align project expectations, and speed up research progress, thus promoting effective interdisciplinary research across Robotics, Neuroscience, and Psychology.

## Introduction

Interdisciplinary collaborations are becoming an increasingly important ingredient for successful research in many fields (Van Noorden, 2015). Combining the expertise of different disciplines helps to address societal challenges (Beckerle et al., 2019) by bringing more comprehensive perspectives and solutions to pressing global issues. This also holds in Robotics, as the need to develop robots apt for interacting with humans is growing (Breazeal, 2004; Wiese et al., 2017) and is among the ten greatest challenges of Robotics (Yang et al., 2018). To build socially intelligent robots fit for bidirectional exchange with other agents, joining forces with other fields such as Neuroscience and Psychology is paramount. However, initiating collaboration between disciplines might not be straightforward, as these fields have different long-standing research traditions and practices. Here, we share eight lessons we learned from initiating our interdisciplinary project across these three fields within the Cluster of Excellence “Science of Intelligence.”

Our research aims at extracting core principles of human interactions from Neuroscience and Psychology experiments for transferring into robot platforms to build communicative robots fit for social interactions where humans and robots exchange information adapting to the environment and to each other (Kirtay et al., 2020). Upon project initiation, we experienced various challenges due to diversity in our backgrounds, training, and discipline cultures, including: divergent project expectations, lack of common terminology, technical misconceptions, varied research procedures, as well as differences in desired research outlets.

Here, we put forward important principles we distilled when facing these challenges. We emphasize especially the integration with Robotics as a promising direction for innovation and advance in Human Neuroscience. Although other researchers have published insights regarding interdisciplinary research teams, they have either reviewed attributes of established successful teams (Lakhani et al., 2012), proposed frameworks to deal with challenges of interdisciplinary research in general (Wright Morton et al., 2015; Tobi and Kampen, 2018), focused on single aspects of the collaboration (e.g., methodology, Smaldino and O’Connor, 2020), or offered examples from collaborations between other disciplines (e.g., Campbell, 2005). Here, we offer a novel contribution by addressing the challenges of bringing together Robotics, Neuroscience, and Psychology, focusing on the most problematic project phase (i.e., initiation), and provide advice that extends to other collaborations involving technical (e.g., engineering) and human-centered (e.g., psychology) disciplines. Additionally, we provide concrete examples to help other researchers picture common problems and anticipate similar challenges. We encourage scientists establishing research collaborations across Robotics, Neuroscience, and Psychology to capitalize on these principles to make their collaboration smoother and more productive and to spare setbacks and frustration.

## Eight Lessons Learned Through an Interdisciplinary Project at the Intersection of Robotics, Neuroscience, and Psychology

### Lesson 1: Align Project Expectations

When researchers plan to bridge to a new discipline, they will likely start by gathering information about the new field and will form expectations on how fruitful such a collaboration could possibly be. Expectations about what is feasible in research fields where they themselves are not experts may be disproportionate. When scientists eventually actively exchange ideas with colleagues from those disciplines, they may realize that the outcome they envisaged is far from what is achievable. For example, availability of robots able to display predefined repertoires of social behavior (e.g., Pepper and NAO) may lead psychologists and neuroscientists to envisage a certain level of autonomy, flexibility, and variety in robots’ behavior. This may induce the expectation that such robots can engage in smooth interactions with humans. Yet, despite using advanced technologies, robots’ social skills are still quite limited. Conversely, roboticists may assume that a measurement of brain activity will lead to mechanistic models of cognitive functions that could be transferred into robots. In fact, most measurements of brain activity in humans reflect the physiological processes underlying cognitive functions only indirectly. Moreover, although advanced computational models have acquired a high level of precision in reproducing multiple aspects of low-level cognitive processes (e.g., perception; Voulodimos et al., 2018; Rankin and Rinzel, 2019), an accurate computational description of higher-level functions (e.g., complex social perception) is far from being reached. Prior expectations developed by roboticists, neuroscientists, and psychologists may diverge also due to the different levels they refer to (i.e., specific actions vs. complex behavior). It is, therefore, crucial to share expectations early on and re-scale them to a level of complexity that can be achieved by all disciplines.

### Lesson 2: Agree on a Common Goal

Researchers’ goals can be distinct across disciplines as what represents a successful outcome varies across fields. Moreover, what seems interesting for one discipline may appear trivial to another. When starting an interdisciplinary project, a crucial step is to clarify everyone’s goals as much as possible to identify discrepancies and points of convergence. Once these have been identified, definitions of new goals may be needed that integrate these different demands. For example, during our first team meeting, two main goals were put forward: on the one hand, Psychology and Neuroscience collaborators aimed to assess whether the same cognitive mechanisms involved in human-human interaction would be involved in human-robot interaction; on the other hand, roboticists focused on reproducing complex behavior in artificial agents to derive testable hypotheses. This discrepancy reflects the difference between the exploratory character of Robotics’ experiments and the confirmatory nature of Psychology and Neuroscience studies (Floreano et al., 2014). It took us some time to figure out that these individual goals could converge into the common goal of endowing robots with biologically inspired computational models.

### Lesson 3: Discuss and Understand Different Research Practices

We experienced a number of challenges due to different research practices across our fields. While Psychology and Neuroscience share many practices, these might be unfamiliar to roboticists. Similarly, research practices in Robotics might appear unconventional to colleagues from Psychology and Neuroscience. Taking the time to understand the respective research procedures is crucial to envisage *how*, and especially *how fast*, the project will develop.

For example, obtaining Ethics approval is standard practice in Psychology and Neuroscience and is mandatory for studies involving human data collection. Therefore, all research projects require Ethics application and approval before data collection can commence. In Robotics, instead, most studies do not require Ethics approval as experiments are carried out on hardware (e.g., the iCub robot) or software platforms (e.g., the Neurorobotics Platform, Falotico et al., 2017) (although there might be exceptions for studies in human-robot interaction and cognitive developmental Robotics). Preparing Ethics proposals and awaiting their approval might take a considerable time and require revisions. Therefore, all team members shall be well-informed about this step when planning the project timeline.

Another increasingly common practice in Psychology and Neuroscience is pre-registration of studies (e.g., Bakker et al., 2020). This refers to the process of registering methods and analysis plans before a study commences. The purpose is to minimize the opportunity for research malpractice (e.g., data fabrication/selection) and to improve reproducibility of research results (e.g., Ioannidis, 2005; Eklund et al., 2016; Renkewitz and Heene, 2019). Pre-registration has the great advantage of carefully pre-planning various experimental features such as hypotheses, data collection and analysis (Botvinik-Nezer et al., 2020), and exclusion criteria in advance. One disadvantage is that considerable time is needed when starting a project to carefully plan each experimental feature. This might be unfamiliar to roboticists who are ready to start collecting data soon after project ideation. Lately, interest in designing reproducible studies is growing in Robotics as well (Bonsignorio and del Pobil, 2015).

Psychology and Neuroscience put special care to assure maximal experimental control, which will allow drawing sound conclusions. Pre-testing procedures, counterbalancing experimental stimuli, and scripting experimental protocols are just a few examples of steps necessary to avoid experimental confounds, achieve robust results, and draw solid conclusions. Roboticists might be surprised by this obsession for “details” and shall be prepared for anticipating these compulsory procedures and the time they require.

Another common practice in Psychology and Neuroscience implemented to assure good-quality data and minimize experimental design flaws is piloting data acquisition. Piloting refers to a preliminary data collection on a small sample of participants conducted to assess the feasibility of a study and to improve experimental details prior to the full-scale data collection. Although extensive piloting is not required in Robotics, some of its practices are comparable to this process. For example, it is common to validate a new model (e.g., deep learning models for reaching and grasping) on robot simulators before deploying it on an actual robot platform. This way, the researchers can fine-tune model parameters for the actual robot and avoid potential hardware problems during experiments. Agreeing with your team on the importance of the piloting phase is thus advisable.

Finally, to reach sufficient statistical power to reliably detect experimental effects, Psychology and Neuroscience studies often require a large number of participants. Sample sizes are usually calculated through power analysis, which estimates the number of participants required to detect an effect of a certain size. Generally, robotics experiments involve none or just a few participants, especially when assessing the effectiveness of developed demonstrators. As sufficient statistical power is fundamental for sound conclusions, the interdisciplinary team should familiarize with this procedure and consistently adopt it. Larger sample sizes affect the project timeline, as data collection will take longer, especially if access to lab space (e.g., fMRI facilities) is limited because shared with other projects run at the facility.

As many factors may affect the project timeline, it is important that the team discusses what might be a possible starting date and how fast the project is expected to proceed, anticipating possible constraints. For example, although psychologists and neuroscientists may be eager to test robots displaying specific social behaviors, it might take considerable time for roboticists to generate such behaviors on the platform. Here, constraints posed by delivering the research output of one discipline reflect on a minor experimental detail of another.

### Lesson 4: Agree on Terminology

Interdisciplinary research projects inevitably host diverse terminology that plays a non-negligible role at various stages of the project, including grant proposal writing, conducting experiments, analyzing data, and disseminating the results. Agreeing on a common terminology early on will facilitate team communication and thus project success.

For example, our research project investigates how different modalities are integrated to enrich social interaction and communication. At first, it was challenging for us to understand what “modality” refers to, as the term has different meanings across our fields. In Robotics, this term indicates the type of sensory data associated with different aspects of the observed phenomenon, such as depth and color data recorded by sensors in an object-recognition experiment (see Ramachandram and Taylor, 2017). However, in Psychology and Neuroscience, “modality” refers to a sensory system (e.g., vision and touch). Thus, in robotic studies, color and depth of an object refer to two different modalities, albeit they are perceived through the same sensory system in biological agents (e.g., the eyes). Note that in Neuroscience modality has yet another meaning as it also refers to the measurement technique (e.g., fMRI or PET).

One way to establish a common terminology is to develop a project-specific dictionary to preserve the project know-how for future team members. Reading project members’ previous publications and gaining knowledge of their respective fields is necessary to identify conflicting concepts and terms whose meaning needs to be agreed upon for effective communication. Mutual understanding in interdisciplinary teams improves with the detail and precision of the communication. Researchers should not assume common knowledge nor be afraid of repeating themselves; redundancy is helpful in interdisciplinary projects to understand each other.

### Lesson 5: Get on the Same Page

Nowadays, still a negligible number of truly interdisciplinary degrees are offered. In most cases, interdisciplinary projects bring together experts from different disciplines who are knowledgeable about different topics and are familiar with disparate literature. Therefore, establishing a functional ground of shared knowledge may be challenging. What helped us immensely in the first months of our interdisciplinary project, was not only sharing literature and discussing research ideas but also jointly engaging in a literature review project. Through this review article (Kirtay et al., 2020), we were able to converge our perspectives and discuss the main findings of our respective fields. Not only were we able to build upon our expertise from three different fields, but we were also able to familiarize ourselves with the key findings of our colleagues’ respective disciplines and develop a shared vision for the project. Finally, it provided us with the chance to develop a good dynamic for working together early on. Hence, engaging in a common literature review project might also help others to get on the same page with literature, key research findings, and form a shared long-term vision for their project.

### Lesson 6: Transfer Essential Technical Knowledge

Overcoming technical challenges is a critical factor to obtain results and finish projects on time. Working with robots in interdisciplinary experiments might pose additional challenges. Sharing essential technical knowledge is crucial to minimize them. Technical aspects of the platform and their official documentation should be introduced to colleagues without a robotics background. Safety-related information, such as handling and cleaning of the robot, monitoring of charging level, the meaning of the light-emitting diodes are also important, as robot misuse could harm both people and itself.

The procedure for generating robot behavior (e.g., processing visual stimuli for object recognition) should be presented step-by-step, including technical details: charging levels, communication protocols, software interface, etc. The robot skills, such as dexterous manipulation, should be illustrated with simple demos. For example, robots’ pointing, reaching, and grasping skills could be displayed through a small-scale experiment where the robot groups objects on the table. These demos should describe the robot sensors (e.g., cameras, touch sensors) employed to carry out the experiments. Basic information on robot control, data processing, and simple troubleshooting should be also provided to the project partners.

Similarly, technical knowledge from the complementary fields should be transferred to roboticists. For example, when planning interdisciplinary experiments that use functional magnetic resonance imaging sharing safety-related information is crucial. A description of practical limitations that may affect experimental design is also necessary. For example, highlighting the need for minimizing movements inside the scanner is important as this constrains what type of tasks can be performed.

Knowledge transfer allows collaborators to correct erroneous expectations and to plan feasible experiments. It should happen on a basic level that enables the partners to understand relevant functioning principles and to anticipate and handle potential issues when running their experiments. The challenges introduced here are just illustrative; setting up novel experiments brings always unknown challenges. However, sharing essential knowledge in advance reduces potential issues before, during, and after the experiments.

### Lesson 7: Agree on the Desired Research Outlets

Different publication venues appeal unequally to different disciplines. In Robotics, conference proceedings are a preferred way to disseminate research. Papers submitted therein undergo rigorous peer-reviewing. These usually have higher impact than journal publications (e.g., Meyer et al., 2009) and are often preferential reads in Robotics. Dissemination at conferences provides more visibility to early-career researchers and has greater impact, as engagement of attendees is high, promoting collaborations with researchers from different Robotics subfields.

At Psychology and Neuroscience conferences, researchers typically present their newest (and usually preliminary) results. Abstracts submitted to conferences do not commonly undergo extensive peer-review as happens for journal submissions. As a consequence, a journal publication in these fields is more valuable, as reflected by higher Impact Factors for journals as compared to conferences. Although this is a questionable metric (Paulus et al., 2018; Larivière and Sugimoto, 2019), it is still widely used in academic evaluations (Else, 2019).

The time necessary to disseminate research outputs is also an important factor to consider when choosing a proper publication venue. To provide robust, reliable results and make the generalizable claims required for journal articles, running additional experiments may be necessary. This adds to the lengthy review-revision cycle that takes at best several months. Instead, conference papers are usually short, present a single study, and the review process is completed within a few months.

Identifying a proper publication venue for interdisciplinary research may carry additional complications. For example, brief research reports of interdisciplinary experiments are generally welcomed by both disciplinary and interdisciplinary conferences. In our experience, such a format is more often unsuccessful when targeting field-specific journals, which are inclined to consider interdisciplinary studies for publication only when submitted as lengthy manuscripts with detailed descriptions and simplified prose.

To manage the expectations of publishing the results of interdisciplinary experiments, the project partners should openly discuss the possible publication venues to balance interests of colleagues from different disciplines. For example, psychologists and neuroscientists may prefer not to publish research results at conferences, as some journals do not consider work already published elsewhere. However, publication of preliminary results at conferences often does not violate this requirement, therefore it is desirable for all counterparts. To accommodate the wishes of the project partners, they should all agree on the publication strategy, ideally at an early stage of the project.

### Lesson 8: Diversity Is an Asset

Interdisciplinarity brings diversity, which is an asset for teamwork. People from different fields likely develop different skills during their careers that could come in handy in joint projects. For example, researchers with a computer science background are usually fluent in programming while psychologists are typically less so. On the other end, psychologists and neuroscientists are typically more trained in experimental design and advanced statistics than roboticists are. Therefore, interdisciplinary teams span a broader range of skills that can be combined to overcome setbacks more effectively.

Additionally, interdisciplinary teams are often more diverse in terms of cultures, genders, and personalities. Such diversity further enriches the research collaboration by bringing in different perspectives and improving problem-solving, flexibility, and innovation of the team (see Schrouff et al., 2019). For example, during our first project retreat, people from fields with unequal gender proportions engaged in common projects. Different gender representations emerged, producing unbalanced communicative exchanges. To promote inclusive discussions, later we made moderation of the debate mandatory for each talk. This measure encouraged participation and facilitated the emergence of different perspectives and, eventually, of innovative ideas.

## Summary and Conclusion

Interdisciplinary collaborations can seem challenging at first, but when collaborators are informed about intricacies of the contributing fields and varied research practices, they can become highly rewarding and can significantly enrich the project and the research field (e.g., Rognini and Blanke, 2016). We believe the eight lessons we presented here will help researchers with a smooth initiation and implementation of projects at the intersection of Robotics, Neuroscience, and Psychology, thus promoting effective interdisciplinary research across these fields.

## Data Availability Statement

The original contributions presented in the study are included in the article/supplementary material, further inquiries can be directed to the corresponding author/s.

## Author Contributions

OW, MK, and DP conceptualized the manuscript and wrote the original draft of the manuscript. DP and AK provided supervision. DP administered the project. RA, J-DH, and VH acquired funding. All authors contributed to manuscript revision and editing, and approved the submitted version.

## Conflict of Interest

The authors declare that the research was conducted in the absence of any commercial or financial relationships that could be construed as a potential conflict of interest.
